# Novel Insights into Food Flavor Chemistry and Analysis

**DOI:** 10.3390/foods15132380

**Published:** 2026-07-03

**Authors:** Roberta Foligni, Gianluca Tripodi

**Affiliations:** Department of Human Sciences and Promoting of the Quality of Life, San Raffaele Telematic University Rome, Via Val Cannuta 247, 00166 Rome, Italy

Aromatic fingerprints provide essential insights into the compositional and structural characteristics of food matrices, as well as into the technological processes and treatments to which they have been subjected. In particular, the volatile compound profile associated with a given food product can be effectively exploited as a multifactorial indicator of quality attributes and shelf-life stability. Furthermore, such profiles represent a robust analytical tool for authenticity and traceability assessment [1,2,3,4], similarly to the lipid and protein profile, enabling the detection of adulteration, mislabeling, or process-induced alterations [5,6,7,8]. Beyond their analytical relevance, volatile fingerprints also constitute a key determinant of sensory perception and consumer acceptance, thereby exerting a direct influence on product marketability and associated economic value [9,10,11]. Chemical characterization of volatile fractions, when integrated with both conventional analytical methodologies (e.g., gas chromatography-based techniques) [12,13] and advanced instrumental approaches (e.g., high-resolution mass spectrometry and sensor-based systems) [14,15], plays a pivotal role in elucidating the effects of innovative processing technologies, alternative preservation strategies, and formulation changes [16,17]. This integrated analytical framework is particularly relevant for evaluating the impact of novel treatments, processing parameters, product development strategies, storage conditions, and the incorporation of emerging or functional ingredients [18,19,20]. Within this context, real-time and dynamic monitoring of volatile compound evolution has emerged as a particularly advantageous approach [21,22,23,24]. Such strategies enable continuous or time-resolved assessment of aroma-related changes, providing a more comprehensive and representative characterization of food systems throughout their entire transformation and storage lifecycle. Consequently, these methodologies support a deeper understanding of process, structure, function relationships in complex food matrices, facilitating improved control over product quality, stability, and sensory attributes [25]. Ponnampalam et al. (2025) examine how production systems, feeding strategies, and processing influence volatile organic compound (VOC) profiles, creating distinct aroma “fingerprints” in animal products. These fingerprints can serve as indicators of quality, authenticity, and traceability. The review highlights advances in analytical methods such as GC–MS, GC–IMS, and electronic noses and links specific VOCs to sensory attributes like grassy, nutty, buttery, or rancid notes that shape consumer perception and willingness to pay. The authors show that these aroma patterns reflect production and processing differences and can support regulatory claims, provenance verification, and label integrity. The volatile profile can also be an indicator of applied agricultural practices that are reflected in the fingerprint of the final product. *Antrodia cinnamomea* is a rare medicinal fungus with notable hepatoprotective, hypoglycemic, and antitumor properties. Ma et al., 2025 compared volatile profiles of its mycelium produced via solid-state (SAC), liquid (LAC), and dish (DAC) cultivation using electronic sensory systems (E-tongue and E-nose), GC-IMS, and multivariate statistical analyses. Distinct aroma profiles were observed among cultivation methods. GC-IMS identified 75 volatile compounds, mainly esters, alcohols, and ketones, with 41 key markers discriminating the groups via PLS-DA and OPLS-DA analyses. These results highlight cultivation-dependent metabolic differences and provide a volatile fingerprint useful for quality control, standardization, and functional food development. Nowadays, the maximum potential in the research and determination of volatile components is offered by the coupling of multiple analytical techniques. Cai et al., 2025 systematically compared the flavor profiles of three traditional Chinese sausages (Cantonese, Five-Spice, and Mala) using HS-GC-IMS, amino acid analysis, electronic sensory analysis, and sensory evaluation. Sensory evaluation showed distinct flavor profiles: Mala sausage was characterized by strong pungency and numbing sensations, Cantonese sausage by sweet and alcoholic notes, and Five-Spice sausage by a milder taste. A total of 39 volatile compounds were identified, with 2-methyl-1-butanol, 2-butanone, and butanal as the most abundant. OPLS-DA highlighted key differential compounds, including terpenes and aldehydes, as major contributors to flavor differentiation. Mala sausage also exhibited significantly higher free amino acid levels, particularly glutamic acid and proline, which strongly influenced taste perception. Overall, the results provide insights into the chemical and sensory basis of flavor variation in traditional Chinese sausages, supporting improved flavor control and product optimization in sausage production. An important step in aromatic characterization is represented by the correlation between data and consumer perception. Tripodi et al., 2025 evaluated monofloral honey from *Capparis spinosa* L. produced by *Apis mellifera sicula* in the Aeolian Islands, focusing on its volatile profile, sensory properties, and consumer acceptance. GC–MS analysis identified 59 volatile compounds, with dimethyl sulfide as the main component. Sensory evaluation revealed a distinctive profile characterized by sweet-caramel, cabbage-like, and pungent notes, which correlated with specific VOCs. Consumer testing showed lower overall preference compared to multifloral honey, although responses varied by age. Despite limited consumer acceptance, the honey’s unique chemical and sensory characteristics indicate potential for niche markets and highlight its relevance for diversification in climate-resilient apiculture. A contribution to be highlighted in the formation and complexity of the generation of the final volatile profile of a food product is given by the storage conditions. Jesenko et al., 2025 investigated the effects of continuous ethanol and hexanal exposure on aroma volatile production in ‘Gala’ apples during six months of controlled atmosphere storage. Apples were stored at 2 kPa O_2_ and 1 °C with either ethanol or hexanal (50 µg L^−1^). Twenty-five volatile compounds were identified, with nine key contributors to aroma. Hexanal increased hexyl acetate, while ethanol enhanced 2-methylbutyl acetate and ethyl 2-methylbutanoate; both treatments promoted 1-butanol formation, particularly after two months of storage. The effects were stronger at mid-storage than after six months, suggesting a temporal decline in precursor effectiveness. Overall, ethanol and hexanal altered aroma biosynthesis pathways and improved the formation of key aroma compounds. These treatments may help preserve or enhance sensory quality in aroma-sensitive cultivars like ‘Gala’ during short- to medium-term storage. Comprehensive New Insights Mechanisms and Detection Methods can provide a comprehensive framework also regarding the senses and in particular the Sweet Taste Transmission. Sun et al., 2025 summarizes current knowledge on sweet taste mechanisms and detection methods, including sensory analysis, electronic tongues, and biosensors, highlighting their advantages and limitations. It also emphasizes recent advances in biosensors based on receptor–ligand recognition and nanomaterials for improved sensitivity and selectivity. Finally, an integrated detection approach combining molecular sensing, data fusion, and artificial intelligence is proposed to enable more precise sweetness evaluation and support the development of healthier food systems. The role of taste has been used in varietal discrimination, specifically for evaluating whether taste alone can distinguish Chardonnay from non-Chardonnay wines. Seinforin et al., 2025 show that Initial discrimination tests indicated that some Chardonnay wines could be identified based solely on taste; however, overall performance was limited compared to conditions including olfaction, confirming the dominant role of aroma in wine recognition. A second set of experiments using the Rate-All-That-Apply (RATA) method identified key taste descriptors such as fattiness, saltiness, bitterness, and acidity as contributing factors in differentiating Chardonnay from other varietals, particularly Sauvignon Blanc. Multisensory evaluation remains essential, although taste attributes still offer valuable insights into wine characterization.

In conclusion, determining and characterizing the volatile profile and combining instrumental analytical techniques with innovative technologies can provide a wealth of information about a food product. The processed data can identify a specific product, but it can also distinguish between different varieties and potential adulterations. Finally, the combination with sensory evaluation and consumer acceptance also provides the basis for marketing aspects that can enhance the final product.

## Data Availability

Not applicable.

## References

[1] Ellis D.I., Brewster V.L., Dunn W.B., Allwood J.W., Golovanov A.P., Goodacre R. (2012). Fingerprinting food: Current technologies for the detection of food adulteration and contamination. Chem. Soc. Rev..

[2] Cuadros-Rodríguez L., Ruiz-Samblás C., Valverde-Som L., Pérez-Castaño E., González-Casado A. (2016). Chromatographic fingerprinting: An innovative approach for food ‘identitation’ and food authentication–A tutorial. Anal. Chim. Acta.

[3] Laurent D. (2017). Food Traceability and Authenticity Based on Volatile Compound Analysis. Food Traceability and Authenticity.

[4] Esteki M., Shahsavari Z., Simal-Gandara J. (2020). Gas chromatographic fingerprinting coupled to chemometrics for food authentication. Food Rev. Int..

[5] Croxton R.S., Baron M.G., Butler D., Kent T., Sears V.G. (2010). Variation in amino acid and lipid composition of latent fingerprints. Forensic Sci. Int..

[6] Dou X., Zhang L., Yang R., Wang X., Yu L., Yue X., Ma F., Mao J., Wang X., Zhang W. (2023). Mass spectrometry in food authentication and origin traceability. Mass Spectrom. Rev..

[7] Torres-Cobos B., Nicotra S.B., Rovira M., Romero A., Guardiola F., Tres A., Vichi S. (2025). Meeting the challenge of varietal and geographical authentication of hazelnuts through lipid metabolite fingerprinting. Food Chem..

[8] Tavoletti S., Foligni R., Mozzon M., Pasquini M. (2018). Comparison between fatty acid profiles of old and modern varieties of *T. turgidum* and *T. aestivum*: A case study in central Italy. J. Cereal Sci..

[9] Mahmud M.C., Shellie R.A., Keast R. (2020). Unravelling the relationship between aroma compounds and consumer acceptance: Coffee as an example. Compr. Rev. Food Sci. Food Saf..

[10] Ye Z., Shang Z., Li M., Qu Y., Long H., Yi J. (2020). Evaluation of the physiochemical and aromatic qualities of pickled Chinese pepper (Paojiao) and their influence on consumer acceptability by using targeted and untargeted multivariate approaches. Food Res. Int..

[11] Nunes M.C., Ferreira J., Raymundo A. (2023). Volatile fingerprint impact on the sensory properties of microalgae and development of mitigation strategies. Curr. Opin. Food Sci..

[12] Gulimeikereyi T., Nurpida A., Dilinuer M., Alai H. (2017). Gas chromatography-based fingerprinting and chemical pattern recognition of Rosa multiflora volatile oil. J. Agric. Sci. Technol. A.

[13] Aulia H.R., Syarifah N., Fitriyani I., Hermansyah H.D., Prastono H. (2025). The Volatile Compound Profile in Edible Oil: A Systematic Review of Gas Chromatography Techniques and their Food Applications. ES Food Agrofor..

[14] Lin H., Jiang H., Adade S.Y.S.S., Kang W., Xue Z., Zareef M., Chen Q. (2023). Overview of advanced technologies for volatile organic compounds measurement in food quality and safety. Crit. Rev. Food Sci. Nutr..

[15] Ahmad A., Diaz F.J., Sendra S., Lloret J. (2026). Machine Learning Unlocks Chemometric Profiling of Plant-Derived Metabolites Using Spectral and Gas Sensor Fingerprints. Food Anal. Methods.

[16] Mannozzi C., Foligni R., Mozzon M., Aquilanti L., Cesaro C., Isidoro N., Osimani A. (2023). Nonthermal technologies affecting techno-functional properties of edible insect-derived proteins, lipids, and chitin: A literature review. Innov. Food Sci. Emerg. Technol..

[17] Tripodi G., Cappelli A., Ferluga M., Dima G., Zaninelli M. (2024). GC-MS and sensory analysis of aqueous extracts of monovarietal American hops, produced using the Synergy Pure™ extraction technique. Foods.

[18] Mannozzi C., Foligni R., Scalise A., Mozzon M. (2020). Characterization of lipid substances of rose hip seeds as a potential source of functional components: A review. Ital. J. Food Sci..

[19] Orkusz A., Rampanti G., Michalczuk M., Orkusz M., Foligni R. (2024). Impact of Refrigerated Storage on Microbial Growth, Color Stability, and pH of Turkey Thigh Muscles. Microorganisms.

[20] Tripodi G., Condurso C., Cincotta F., Merlino M., Verzera A. (2020). Aroma compounds in mini-watermelon fruits from different grafting combinations. J. Sci. Food Agric..

[21] Li T., Song F., Bai Y., Wu F., Ruan M., Cao Y., Zhou L., Sun F. (2023). Real-time emission, chemical properties, and dynamic evolution mechanism of volatile organic compounds during co-pyrolysis of rice straw and semi-bituminous coal. ACS EST Eng..

[22] Han Z., Ahmad W., Rong Y., Chen X., Zhao S., Yu J., Zheng P., Huang C., Li H. (2024). A gas sensors detection system for real-time monitoring of changes in volatile organic compounds during oolong tea processing. Foods.

[23] Lin H., Ji M., Huang B., Kwadzokpui B.A., Adade S.Y.S.S., Chen Q. (2026). Overview of Electronic Olfactory Systems for Volatile Compound Measurements During Food Processing: Principles, Methods, Systems, and Applications. J. Food Process Eng..

[24] Ablegue A.G.M.D., Zhang M., Guo Q., Rui L. (2026). Monitoring and shelf life extension of food products based on machine learning and deep learning: Progress and potential applications. Food Rev. Int..

[25] Costa S.P., Cardoso A., Mahmoodnia H., Gonçalves F., Miranda A., Yamada F., Guimarães L., Barbosa F., De Beule P. (2026). Bacterial species differentiation via real-time detection of microbial volatile organic compounds using a wavelength multiplexed photoionization detector and AI image-based analysis. Sci. Rep..

